# Zebrafish knockout of Down syndrome gene, *DYRK1A*, shows social impairments relevant to autism

**DOI:** 10.1186/s13229-017-0168-2

**Published:** 2017-09-29

**Authors:** Oc-Hee Kim, Hyun-Ju Cho, Enna Han, Ted Inpyo Hong, Krishan Ariyasiri, Jung-Hwa Choi, Kyu-Seok Hwang, Yun-Mi Jeong, Se-Yeol Yang, Kweon Yu, Doo-Sang Park, Hyun-Woo Oh, Erica E. Davis, Charles E. Schwartz, Jeong-Soo Lee, Hyung-Goo Kim, Cheol-Hee Kim

**Affiliations:** 1 Department of Biology, Chungnam National University, Daejeon, 34134 Republic of Korea; 2 Korean Research Institute of Biosciences and Biotechnology, Daejeon, 34141 Republic of Korea; 3 Department of Functional Genomics, Korea University of Science and Technology, Daejeon, 34113 South Korea; 4 Center for Human Disease Modeling, Duke University Medical Center, Durham, NC 27701 USA; 5 Greenwood Genetic Center, Greenwood, SC 29646 USA; 6 Dementia DTC R&D Convergence Program, Korea Institute of Science and Technology, Seoul, 02792 South Korea; 7 Department of OB/GYN, Department of Neuroscience and Regenerative Medicine, Augusta University, Augusta, GA 30912 USA

**Keywords:** Autism, Down syndrome, Knockout, Zebrafish, *DYRK1A*, Social interaction, Shoaling, Group behavior

## Abstract

**Background:**

*DYRK1A* maps to the Down syndrome critical region at 21q22. Mutations in this kinase-encoding gene have been reported to cause microcephaly associated with either intellectual disability or autism in humans. Intellectual disability accompanied by microcephaly was recapitulated in a murine model by overexpressing *Dyrk1a* which mimicked Down syndrome phenotypes. However, given embryonic lethality in homozygous knockout (KO) mice, no murine model studies could present sufficient evidence to link *Dyrk1a* dysfunction with autism. To understand the molecular mechanisms underlying microcephaly and autism spectrum disorders (ASD), we established an in vivo *dyrk1aa* KO model using zebrafish.

**Methods:**

We identified a patient with a mutation in the *DYRK1A* gene using microarray analysis. Circumventing the barrier of murine model studies, we generated a *dyrk1aa* KO zebrafish using transcription activator-like effector nuclease (TALEN)-mediated genome editing. For social behavioral tests, we have established a social interaction test, shoaling assay, and group behavior assay. For molecular analysis, we examined the neuronal activity in specific brain regions of *dyrk1aa* KO zebrafish through in situ hybridization with various probes including *c-fos* and *crh* which are the molecular markers for stress response.

**Results:**

Microarray detected an intragenic microdeletion of *DYRK1A* in an individual with microcephaly and autism. From behavioral tests of social interaction and group behavior, *dyrk1aa* KO zebrafish exhibited social impairments that reproduce human phenotypes of autism in a vertebrate animal model. Social impairment in *dyrk1aa* KO zebrafish was further confirmed by molecular analysis of *c-fos* and *crh* expression. Transcriptional expression of *c-fos* and *crh* was lower than that of wild type fish in specific hypothalamic regions, suggesting that KO fish brains are less activated by social context.

**Conclusions:**

In this study, we established a zebrafish model to validate a candidate gene for autism in a vertebrate animal. These results illustrate the functional deficiency of *DYRK1A* as an underlying disease mechanism for autism. We also propose simple social behavioral assays as a tool for the broader study of autism candidate genes.

**Electronic supplementary material:**

The online version of this article (10.1186/s13229-017-0168-2) contains supplementary material, which is available to authorized users.

## Background

ASD is a genetically and clinically heterogeneous group of neurodevelopmental disorders representing various subtypes of altered social communication, unusually restricted interests, or repetitive behavior [1]. Next-generation sequencing approaches have identified additional nonsense, frameshift, and insertion/deletion mutations in ASD or intellectual disability cases [2–4].

In humans, *DYRK1A* is located on chromosome 21q22.13 in the “Down Syndrome Critical Region (DSCR)” at 21q22.1–q22.3 [5]. This gene has been proposed as a major contributor to the pathogenesis of Down syndrome, Alzheimer’s disease, and Huntington’s disease [6–8]. However, truncation of *DYRK1A* due to balanced chromosome translocations was previously reported in two unrelated individuals with overlapping phenotypes of developmental delay and microcephaly [9]. Subsequently, mutations in *DYRK1A* are also associated with primary microcephaly, intellectual disability, and ASD [10–13]. In this report, we describe a newly affected individual with a heterozygous 21 kb intragenic deletion which involves the last five exons of *DYRK1A*; the individual exhibits ASD in addition to learning difficulties and microcephaly.

Since these distinct cognitive phenotypes could arise from either increase or decrease of gene dosage, overexpression and KO techniques of *DYRK1A* were applied to animal models in order to elucidate the underlying mechanism. Intellectual disability coupled with microcephaly was recapitulated in a *Dyrk1a* overexpressing murine model which mimicked Down syndrome patients who possess an extra copy of chromosome 21 [14, 15]. *Dyrk1a* null mutants exhibit generalized growth delay, including an overall reduction in the size of the developing brain as well as embryonic lethality during mid-gestation [16–18]. Heterozygous mutants show decreased neonatal viability and reduced brain size from birth to adulthood. Neurobehavioral analysis revealed that heterozygous mutants in adulthood are deficient in motor function and learning [18–20]; however, none of these murine model studies present sufficient evidence to directly link *Dyrk1a* dysfunction with autism in the context of social interaction of an ASD animal model.

To understand the molecular mechanisms underlying microcephaly and ASD, we established an in vivo KO model using zebrafish. The zebrafish (*Danio rerio*) is a tractable vertebrate model in biological research, especially in the fields of neuroscience [21, 22]. Recent scientific reports show conservation of brain structures between zebrafish and humans, such as the amygdala, hippocampus, habenula, and hypothalamus [23]. Moreover, *Danio rerio* displays broad complex behaviors in aspects of learning, cognition, aggression, anxiety, and social interaction [22]. The zebrafish and human genomes are well conserved with more than 80% of human disease genes represented in the zebrafish model [24]. Thus, the zebrafish is a useful tool in elucidating the function of novel genes involved in head formation or neurogenesis [25, 26] and, more recently, for validating the function of human candidate genes involved in microcephaly, intellectual disability, and ASD [27–31].

We employed targeted KO of the zebrafish *DYRK1A* orthologue and found that *dyrk1aa* KO zebrafish exhibits microcephaly and impaired social behavior which is a key representative feature of ASD. Also, we report on the development of two approaches in assessing behavioral phenotypes of the zebrafish ASD model. Since social behavioral analysis in the context of ASD has not been reported on any other *DYRK1A* animal model, we undertook the analysis of social and group behavioral interactions in the *dyrk1aa* KO zebrafish. Several social interaction tests have already been addressed which assess the social behavior of zebrafish [32, 33]; however, we have improved upon these social interaction assays by newly developing the shoaling bowl assay in which a flat-round bowl provides a convenient means for assessing group behavior in zebrafish autism models.

## Methods

### Clinical report

The proband was noted as being small for gestational age according to regular ultrasound scans. The affected female of northern European ancestry, now age 11 and half years, was born at 37-week gestation by emergency Cesarean section due to a drop in heart rate. Her birth weight was 1.9 kg. Due to breathing problems around the time of birth, the subject required suction at birth and did not cry. Afterward, she was administered oxygen and housed in the Special Care Baby Unit. The subject had a computed tomography (CT) brain scan at 1 year 3 months which showed mild cerebral atrophy involving mainly the frontal lobes. At age 3 years 1 month, she had a magnetic resonance imaging (MRI) scan and microcephaly was noted. Her head circumference has always been at − 5 standard deviations being below the 0.4th percentile. Her MRI showed increased X-ray CLC spaces which is a reflection of a moderate degree of cerebral volume loss, more so in the white matter than in gray. There were also some abnormal subcortical high signals in both temporal lobes; however, no overlaying abnormality was present. Also, there was evidence of thinning in the corpus callosum as well as a degree of volume loss in the medulla oblongata compared to previous scans. In addition, a mild dilatation of the lateral ventricles probably represented white matter loss. There was a small high-signal area seen in the white matter of the right parietal lobe representing gliosis.

Developmentally, the subject could sit alone at 8 months, roll from front to back by 1 year, commando crawl at 14 months, crawl properly around 16 ½ months, pull to stand at 15 months, walk around furniture at 1 year 7 months, and walk alone at 2 years despite having immature gait. She did not require the need of walking aids, but her legs, hips, knees, and ankles have always been very stiff. The subject’s parents approached their doctor when she was 3 years 6 months old, and she was diagnosed, at 6 years of age, with scoliosis of the back, differing leg lengths, inflexibility, and possible cerebral palsy. She has not had any serious head injuries but started having seizures at about 13 months. At the age of 2 ½, she was diagnosed with epilepsy and continues to have four or five serious seizures a year; each lasts over an hour. At 9 years 6 months, she had increasing difficulty in straightening her knees completely and walking, requiring frequent use of a wheelchair. At her latest clinical assessment, she displayed an increase in tone in her upper extremities and continues to be ambulatory. Clinically, her spinal deformity has not worsened as confirmed by full spine X-rays. She has a limb length discrepancy—shorter on the left than the right—with some pelvic obliquity. She also has bilateral valgus ankle joints and recurrent chest infections.

The stiffness in her arms and wrists makes dressing and undressing difficult. She has a combination of diagnoses including global development delay, ASD, learning difficulties, and illiteracy. Also, her level of speech at 9 years 8 months of age was that of a 3- to 4-year-old and required attendance to a special school. She continues to present high levels of challenging behaviors associated with distress and anxiety, continued problems in sociability, and little interest in her peers preferring to play on her own. She does not like crowds and might kick and shout at people if they invade her personal space. While she can be clingy to her parents, she makes very little eye contact and is fixated on particular items such as footballs and goggles; the latter of which she has at least 17 pairs that she wears at home but never at the swimming pool. She flaps her hands when excited, and she continues to have problems with attention and decreased concentration.

### Microarray analysis

Array comparative genomic hybridization (CGH) was carried out using a BlueGnome 8x60k International Standard Cytogenomic Array (ISCA) design oligonucleotide microarray. Test DNA was referenced against same-sex control DNA, and data was analyzed in BlueFuse Multi v2.2. This platform should detect the majority of copy number imbalances > 15 kb in 500 disease gene/telomeric regions and > 180 kb in the genomic backbone and may detect smaller imbalances in some instances. The derivative log ratio (DLR) quality score given for this hybridization is 0.21. Probes are mapped to GRCh37/hg19.

### Generation of *dyrk1aa* KO zebrafish

We identified the zebrafish *dyrk1aa* gene and its exon/intron boundaries by searching the Ensembl database (GRCz10 Ensembl gene ID: ENSDARG00000063570; transcript ID: ENSDART00000100073). The *dyrk1aa* (7 bp deletion) KO fish was generated using TALEN, as previously reported [34]. A TALEN pair targeting exon 5 of *dyrk1aa* (left target site: 5′-tgg gtc gcc atc aag atc at-3′; right target site: 5′- gcc ttc ctg aat cag gct ca-3′) was designed and assembled by ToolGen Inc. (http://toolgen.com/). In vitro*-*transcribed RNA of the TALEN pair (100 ng each) was microinjected into 1~2 cell stage of fertilized zebrafish eggs, which were then grown to 4-month-old adulthood. A stable mutant line, *dyrk1aakrb1*, was identified and genotyped by direct PCR and sequencing performed using two sets of nested primers: the outer primer pair 5′-cca gca aca aga agg aga gg-3′ (forward) and 5′-agc cct gat ctt tcc agg tt-3′ (reverse) and the inner primer pair 5′-tta caa cga cgg cta tga cg-3′ (forward) and 5′-ttc atc tcg gtg tcg tgc t-3′ (reverse). The PCR amplification conditions were as follows: for primary PCR, 35 cycles of 95 °C, 20 s; 59 °C, 40 s; 72 °C, 1 min; and for secondary PCR, 25 cycles of 95 °C 20 s; 55 °C, 40 s; 72 °C, 30 s. The progeny were propagated through a series of out-crossings with wild type (WT) fish; these animals were eventually in-crossed to obtain homozygous KOs. The KO zebrafish line is deposited in the KCTC (http://biorp.kribb.re.kr/) with deposit number, BP1294898.

### Brain histology and expression analysis

To ascertain brain histology, 7-month-old male WT and KO fish were fixed in 4% paraformaldehyde (PFA) solution overnight, then compared for body length. Among fish of the same size and age, brains were isolated and imaged and sizes were measured using ImageJ software. After dehydration in ethanol and clearing in xylene, brains were infiltrated with paraffin, embedded, and serial-sectioned. The sections (10-um thick) were stained with hematoxylin-eosin. The total area and ventricle area of the brain in the sections were measured using ImageJ and the ratio (ventricle area/total area ×100) was calculated. In situ hybridization was performed as previously described [35] using the following digoxigenin (DIG RNA labeling kit, Roche)-labeled antisense probes: *sox2*, *neurog1*, *ccnd1*, *c-fos*, *crh*, *oxt*, *th1*, *vglut2.2*, and *gad1b*. For *c-fos* analysis, 7-month-old male WT and KO zebrafish were fixed in 4% PFA solution immediately after social interaction test. For *crh* analysis, 7-month-old male WT and KO fish were fixed after social isolation. For *oxt*, *th1*, *vglut2.2*, and *gad1b* analysis, 7-month-old male WT and KO fish from their home tank were fixed. To detect cell death, 3-week-old zebrafish larvae were fixed in 4% PFA solution for 4 h at room temperature. Fixed larvae were embedded in agar-sucrose solution (1.5% agar, 5% sucrose). The agar blocks containing the larvae were sunk in 30% sucrose solution and processed for transverse cryostat serial-sectioning. The sections (25-um thick) were immuno-stained with an antibody against activated caspase-3 (BD Biosciences), which marks apoptotic cell death.

### Behavioral tests for early larval zebrafish

#### Dark flash test

Dark flash test was performed as previously reported [36]. Free swimming 6 dpf larvae were placed in a 24-well plate (SPL life Sciences— each well contains a single larva— then inserted into the DanioVision Observation Chamber (Noldus). To induce freezing/startle response, dark flash pulses illuminated the plate for 30 s followed by lights off for 30 s (flash-off dark condition). This scheme was repeated five times. Locomotive response to visual stimuli was measured by video-tracking analysis using EthoVision XT7 software (Noldus). For analysis of locomotor activity, raw data was converted into total distance moved (cm) by each larva per 10 s time-bins. After behavioral assay, each zebrafish larva was genotyped using genomic PCR.

#### Sleep and waking activity

Sleep and waking activity was measured as previously described [37]. *dyrk1aa* KO embryos and control WT embryos were raised in a light- and temperature-controlled incubator. Five-day-old larvae were placed in a 24-well plate in the observation chamber of Danio Vision tracking system for acclimation under controlled lighting conditions (12 h–12 h light-dark cycles). Starting from 5 dpf, locomotion of each larva during day and night phases were tracked and analyzed by EthoVision XT7 software over a course of 2 days. Locomotor activity was analyzed by converting raw data into the velocity (cm/s) of each larva per 30-min time-bins.

### Social and group behavior tests for adult zebrafish

#### Novel tank assay

Novel tank assay was performed as previously described [38]. Each 7-month-old male WT or KO zebrafish was placed in a transparent tank with dimensions measuring 24 × 15 × 15 cm. We replicated the novel tank assay with eight WT and eight KO fish. The back side of the tank was covered with a white sheet to aid data recording. We used a three-compartment novel tank with top, bottom, and middle virtual zones. All behavior tests were recorded for a period of 10 min from the lateral viewpoint of the tank using a video camera (Sony, HDR-CX190). Fish were returned to their home tanks immediately after completion of the test. The raw data was analyzed using EthoVision XT7 software.

#### Social interaction assay

The social interaction test was modified and improved upon from a previous study [32]. The tank was divided into two sections by placing a metal mesh or an acrylic plate separator at the first quarter of the tank. To conduct the social interaction test, the first section of the tank was designated as the social cue. The second section was used as the space to investigate the behavior of tester fish. In every experiment, we used different 7-month-old male fish for both the social cue and tester to maintain similar conditions. We replicated this experiment with 30 WT and 30 KO tester fish, in total. The second section was divided further into four equal sub-zones; the zone nearest to the social cue was designated zone “I”, the second nearest zone “II”, the third zone “III”, and the last zone “IV”. The hollow-rectangular pattern of the metal mesh separator (0.3 × 0.3 cm) created a gray shadow, while the acrylic plate was transparent. All behavioral tests were performed between 13:00 and 17:00 h using water from a tank adjusted to the holding room temperature. All experimental fish were raised in a social environment. One day prior to each test, fish were transferred to a different tank in an isolated environment. All behavioral tests were recorded from the lateral viewpoint of the tank, for a period of 15 min using a video camera.

#### Shoaling bowl assay

Fish form groups in a behavior called shoaling [39–41]. To test whether *dyrk1aa* KO zebrafish show altered shoaling behavior, a group of 7-month-old fish (*n* = 3–7 fish per group) was placed together and monitored by video tracking. We introduced a unique and simple device to test and quantify shoaling behavior. First, we examined several types of bowls (with different shapes, sizes, depths, and colors) and selected a round, flat bottom, white bowl for further experiments (upper half diameter, 33 cm; bottom diameter, 24 cm; height, 11 cm; and water depth, 3.2 cm). All tests for group behavior were recorded for a period of 15 min using a video camera at a fixed height with a top view of the bowl. The recorded videos were analyzed using 31 screenshots made every 10 s for 10–15 min measuring the distances between individual fish in the group using the ImageJ program.

### Statistical analysis

In all experiments, comparisons between WT and KO fish were done using a two-tailed, Student’s *t* test. Data are expressed as mean ± standard error of the mean (SEM). In all tests, *p* < 0.05 was considered to be significant. * indicates *p* < 0.05, ** indicates *p* < 0.01, and ***p < 0.001.

## Results

### Intragenic microdeletion of *DYRK1A* in an ASD patient

**Fig. 1:**
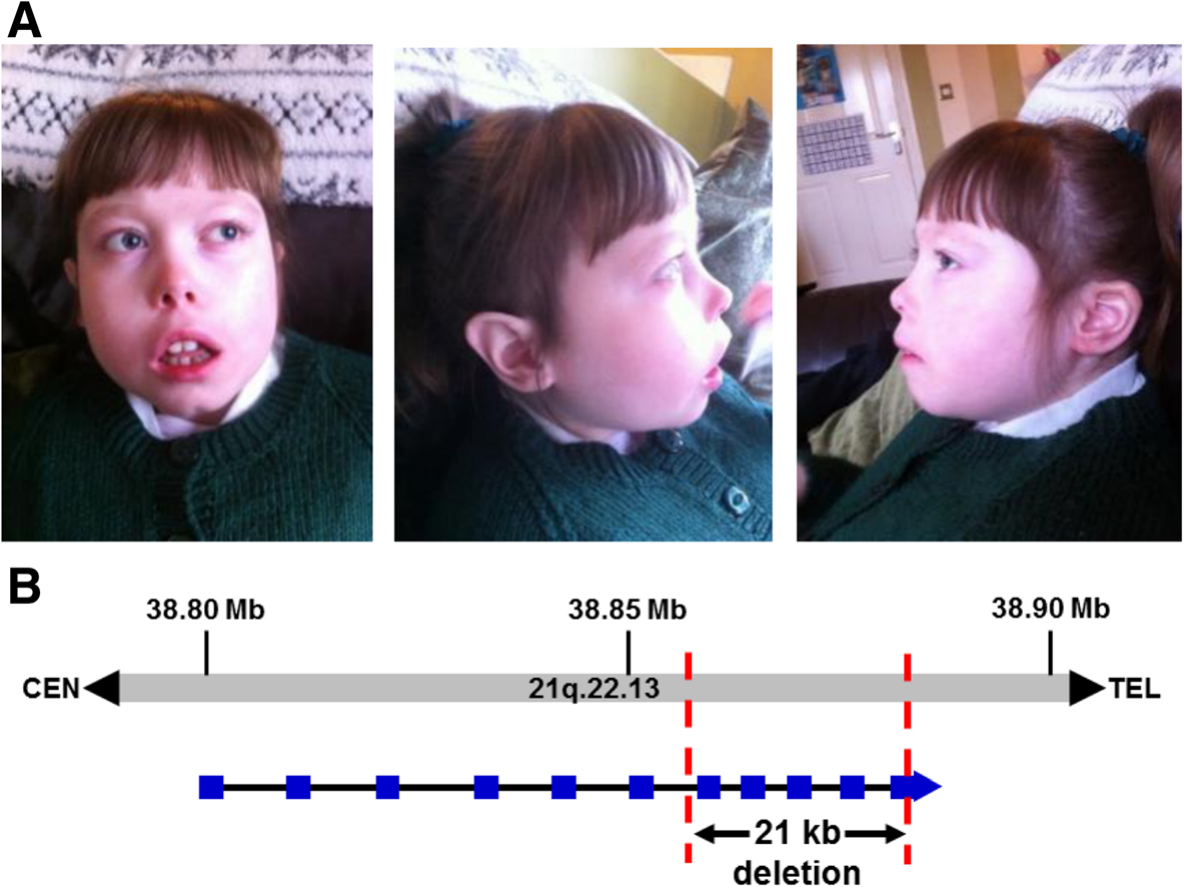
Microarray detected an intragenic microdeletion of *DYRK1A* in an individual with microcephaly and autism. **a** Pictures of the index case, aged 11 years, who displays microcephaly and autism. **b** Schematic of 21q22.13 showing the region of the 21 kb deletion involving the last five exons of *DYRK1A*

Microarray analysis of a patient with ASD and microcephaly was used to detect a de novo 21 kb microdeletion at 21q22.13, arr [hg 19] (38,865,151–38,885,792)X1 dn, within the *DYRK1A* gene (Fig. 1).

### Generation of *dyrk1aa* KO zebrafish

**Fig. 2:**
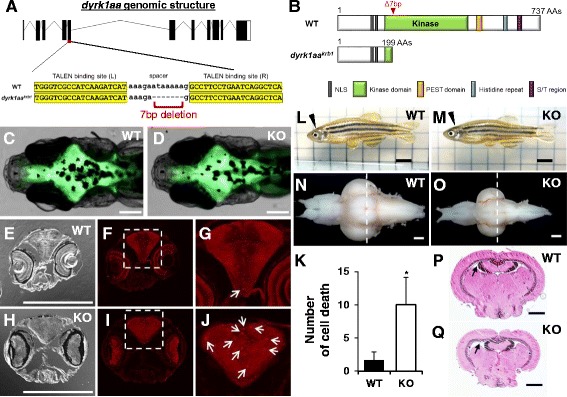
Generation of *dyrk1aa* KO zebrafish and microcephaly phenotype analysis. **a** Schematic representation of the genomic structure of *dyrk1aa* and a 7 bp deletion generated by gene targeting with TALEN. **b** Predicted structures of WT and *dyrk1aa* KO mutant proteins in zebrafish. The 7 bp deletion resulted in a frame-shift mutation and premature termination at the kinase domain. **c**, **d** Similar brain size in WT (**c**) and KO fish (**d**) is seen at the 2-week-old stage. Fluorescent live neurons are visible in the genetic background of *HuC:eGFP* transgenic zebrafish. Anterior to the left, dorsal view. Scale bars 0.2 mm. **e** *–* **j** Sections of a 3-week-old zebrafish head region were immuno-stained with an antibody against activated caspase-3. Coronal sections at the level of the eye: bright-field image (**e**, **h)** and fluorescent image (**f**, **g**, **i,** and **j**). **g**, **j** Magnification of inset in (**f**, **i**). Arrows indicate caspase-3 positive cells in the brain. Scale bars 0.2 mm. **k** The number of caspase-3 positive cells is increased in the brain of KO fish. Five animals for each WT and KO fish were used for the analysis. Data are presented as mean ± SEM. * *p* < 0.05 by Student’s *t* test. **l**, **m** Pictures of adult WT and KO zebrafish. *dyrk1aa* KO zebrafish were normal in body length and overall morphology except for a reduction of brain size. Arrowheads indicate the position of the brain in the head region. Scale bars 5 mm. **n**, **o** Photograph of dissected brains from WT and KO zebrafish, showing microcephaly phenotype in KO zebrafish. Anterior olfactory bulbs were positioned at the left, ventral view. Scale bars 0.4 mm. **p**, **q** Confirmation of microcephaly phenotype in KO zebrafish by histological examination. Dashed line in **n** and **o** indicates the relative section position used in **p** and **q**. Brain sections were stained with H&E. KO zebrafish brain had wider ventricle space than WT zebrafish. Arrows point to TeV. Scale bars 0.4 mm

To model *DYRK1A* dysfunction in zebrafish, we generated a loss-of-function mutant using TALEN-targeted mutagenesis [34]. Genotyping of F0 adults identified three KO zebrafish out of 65 founder fish, with a targeting efficiency of 4.6%. Finally, one stable KO was established as a *dyrk1aa* KO zebrafish line, called *dyrk1aa* ^*krb1*^. This *dyrk1aa* KO line harbors an aberrant early stop codon due to a seven base pair (7 bp) deletion in exon 5 of *dyrk1aa* which likely leads to the truncation of the protein, including most of the kinase domain, and loss of function (Fig. 2a, b).

### Characterization of *dyrk1aa* KO zebrafish at early larval stages

*dyrk1aa* KO zebrafish showed normal development of gastrulation and morphology at early stages. Since *DYRK1A* is known to play key roles in cell proliferation, survival, and differentiation during neurogenesis in mouse models [16, 42], we tested the expression of the neural stem cell marker, *sox2*, and the neuronal determination marker, *neurog1*, by whole-mount in situ hybridization. No significant change of *sox2* and *neurog1* expression were found in the *dyrk1aa* KO embryos at early developmental stages, 24 h post-fertilization (hpf), and 48 hpf (Additional file 1: Figure S1A-D). We also examined the expression of cell cycle marker, *cyclin D1* but found no detectable changes in *dyrk1aa* KO embryos compared to WT (Additional file 1: Figure S1E, F).

Next, we tried to identify any behavioral changes in *dyrk1aa* KO fish at the early larval stage, i.e., 6 days post-fertilization (dpf). Zebrafish eyes begin to detect light by 3.5 dpf, and zebrafish larvae start swimming freely at 5 dpf [36, 43]. We measured the locomotive response to visual stimuli by video-tracking analysis. At 6 dpf, both WT and *dyrk1aa* KO larvae showed similar patterns of response to visual stimuli (Additional file 1: Figure S1G). Also, we examined circadian rhythms of KO larvae by measuring locomotor activity under day-night cycles over a period of 2 days, between 5 and 7 dpf [37]. The zebrafish pineal gland contains a circadian oscillator which drives rhythms of melatonin synthesis and transduction mechanisms for entrainment by light cycles [44, 45]. Quantitative profiling revealed that KO larvae display similar activity during both day and night when compared to controls (Additional file 1: Figure S1H).

### Adult *dyrk1aa* KO zebrafish display microcephaly

*DYRK1A* is known to be involved in brain size regulation in different model organisms, as evidenced by a small brain phenotype upon loss-of-function [18, 46]. Since *dyrk1aa* KO adult zebrafish were not significantly different from WT siblings when assessed for body length and overall morphology (Fig. 2l, m), we examined brain size of *dyrk1aa* KO fish. *dyrk1aa* WT and KO brains were dissected, and KO zebrafish brains were found to be significantly smaller than those of WT (Fig. 2n, o). KO zebrafish brains also had pronounced size reduction in specific brain regions, including the telencephalon (Tel), tectum opticum (TeO), and corpus cerebelli (CCe) (Additional file 2: Figure S2A). Examination of brain sections further supported our morphological analyses. In transverse sections, we found that the space of KO brain ventricles was wider than that of WT brains, especially in the tectal ventricle (TeV) (Fig. 2p, q; Additional file 2: Figure S2B). To identify an intermediate developmental time-point in which neurological phenotypes begin to occur, we assessed *dyrk1aa* KO fish in late larval stages. When brain sections were stained with an antibody against activated caspase-3, known to detect apoptotic cell death [47], we found an increased number of apoptotic cells in the brain of *dyrk1aa* KO fish at 3 weeks of age compared to that of age-matched WT fish (Fig. 2c–j). Together, these results suggest that the microcephaly phenotype may be attributed to neuronal cell death during brain development, which is consistent with data reported by previous studies [47].

### *dyrk1aa* KO zebrafish display anxiolytic behavior in a novel tank assay

**Fig. 3:**
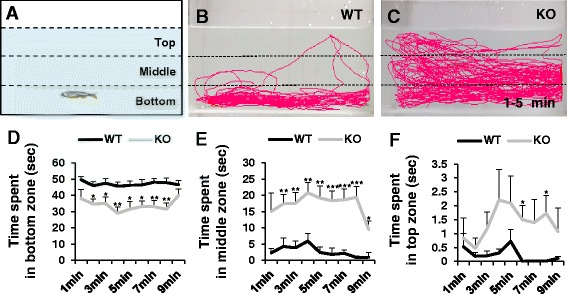
Novel tank assay showing anxiolytic behavior in *dyrk1aa* KO zebrafish. **a** An illustration of a novel rectangular tank. Dashed lines indicate the boundaries of three vertically different zones (top, middle, and bottom). **b**, **c** Representative images of zebrafish behavior in the early time phase (1–5 min). *dyrk1aa* KO zebrafish display reduced anxiety-like phenotype, swimming more time in middle and top zones. **d**–**f** Duration per minute in each zone. Upon introduction to the novel tank, zebrafish display a preference to remain at the bottom zone. *dyrk1aa* KO fish spent significantly less times in this zone, compared to WT siblings. Black line for WT and gray line for *dyrk1aa* KO zebrafish. Number of fish used in this assay: *n* = 8 for WT fish, *n* = 8 for KO fish, respectively. Data are presented as mean ± SEM. * *p* < 0.05, ** *p* < 0.01, *** *p* < 0.001 by Student’s *t* test

*dyrk1aa* KO zebrafish are viable and fertile into adulthood. To examine behavioral changes of adult *dyrk1aa* KO fish, we performed a novel tank assay aimed to measure anxiety. This test is based on the animal’s innate behavior to seek protection in a novel environment by freezing and reducing exploratory behavior [38]. As fish gradually adapt to a novel environment, an increase in exploration usually occurs, which is characterized by (1) increased time spent in the top of the tank, (2) increased entries to the top of the tank, and (3) decreased freezing [48–50]. Using a three-compartment novel tank with top, bottom, and middle zones (Fig. 3a–c), we found that KO fish spent significantly more time in the middle or top zones than in the bottom than WT fish (Fig. 3d, e; Additional file 3: Figure S3A, B). In addition, KO fish displayed less freezing time than WT fish (Additional file 3: Figure S3C); however, there were no significant differences for total distance and speed of movement in both WT and KO fish, indicating that this phenotype is not due to motor deficits (Additional file 3: Figure S3D, E). Taken together, these data suggest that *dyrk1aa* KO zebrafish experience less anxiety than WT fish.

### Establishment of a social interaction assay in WT zebrafish

Social interaction is an essential behavior of zebrafish as they express strong preferences towards conspecifics [32]. We standardized the social interaction test in WT zebrafish and tested the effect of group size of the “social cue” on the behavior of a “tester” fish by increasing the number of fish in a group from one to five (Additional file 4: Figure S4). In most cases, WT tester fish showed a clear preference for the cue fish group by staying in the most proximal zone “I” for a longer period of time than in the more distant zones II, III, and IV. This tendency of social interaction increased gradually according to the group size of the social cue. However, we determined that optimal group size of the social cue was three fish under these experimental conditions. Next, we tested the effect of different separating materials on social interaction. We noted a difference of social interaction between metal mesh and acrylic plate separators (Additional file 4: Figure S4A, C) and speculated better visibility of tester fish towards the social cue with the transparent acrylic plate rather than the metal mesh accounted for the differences. Since metal mesh in the water tank has a dark-gray color (shadow-like) with hole-like patterns, we reasoned that this might interrupt tester fish perception of the social cue. Next, we established a reliable time frame for the measurement of social interaction. After video recording for 15 min, the data corresponding to different time frames were collected for every minute and analyzed. Since animals, including fish, tend to show anxiety-like behavior in new environments and require time for acclimation, we chose the 6–10 min time frame for social interaction analysis. After 10 min, the WT tester fish began to show a diminished degree of social interaction (Additional file 4: Figure S4E, F).

### *dyrk1aa* KO zebrafish show impaired social interaction

**Fig. 4:**
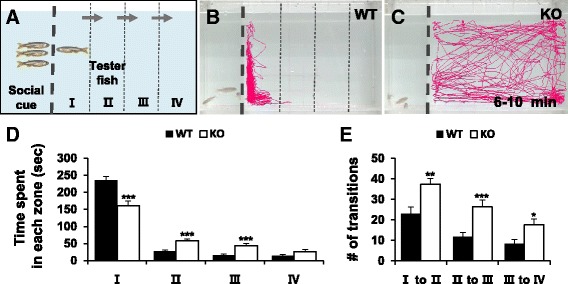
Social interaction assay showing impaired social behavior in *dyrk1aa* KO zebrafish. **a** Here, three fish were used as a social cue against a tester fish. Bold dashed line indicates the position of the separator in the water tank. Three narrow dashed lines indicate the boundaries of four different zones (I, II, III, and IV) in the moving space of the tester fish, ranging from the most proximal to the most distal, respectively from the social cue fish group. Arrows indicate the transition of tester fish between zones. **b**, **c** Video tracking of 5-min movements of WT or *dyrk1aa* KO fish, showing the social interaction with the social cue. **d** Duration time for tester fish in each different zone. Black bars for WT fish and white bars for KO mutant fish. **e** Number of transit movements of tester fish at each zone boundary; movement from “I” to “II”, “II” to “III”, and “III” to “IV” zone. Number of tester fish used in this assay: *n* = 30 for WT fish, *n* = 30 for KO fish, respectively. Data are presented as mean ± SEM. * *p* < 0.05, ** *p* < 0.01, *** *p* < 0.001 by Student’s *t* test

**Fig. 5:**
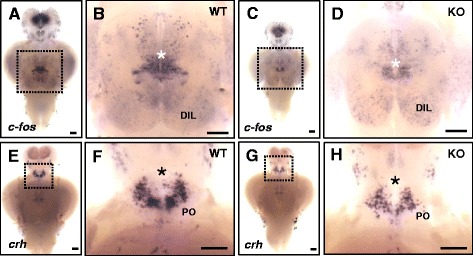
In situ hybridization of *c-fos* and *crh* in the brain of KO and WT fish. **a**–**d** Detection of *c-fos* mRNA expression in the dissected brain of WT (**a**, **b**) and KO (**c**, **d**) fish. Induction of strong *c-fos* expression is seen in specific brain regions (white asterisk) in WT fish (**b**) during social interaction, compared to that in KO fish (**d**). Higher expression of *c-fos* is observed in the diffuse nucleus of the inferior lobe (DIL) in the KO fish brain. fb, forebrain; mb, midbrain; hb, hindbrain; hy, hypothalamus. Ten animals for each WT and KO fish analysis were used. **e**–**h** Detection of *crh* expression in the brain of WT (**e**, **f**) and KO (**g**, **h**) fish. After acute social isolation for 24 h, *crh*-expressing cells are slightly reduced in preoptic area (PO, black asterisk) in KO fish (**h**), compared to that of WT fish (**f**). Eight animals for each WT and KO fish analysis were used. Anterior to the top and ventral view. **b**, **d**, **f,** and **h** Magnification of the ventral hypothalamic region boxed in **a**, **c**, **e,** and **g**. Scale bars 0.2 mm (**a**, **c**, **e**, and **g**), 0.76 mm (**b**, **d**), and 0.94 mm (**f**, **h**)



*DYRK1A* has been associated with ASD in humans by previous reports [12, 13]. In our study, we tested whether the *dyrk1aa* KO zebrafish could be used as an animal model for the study of ASD. After 15 min of video recording (Additional file 5: Figure S5), the 6–10 min time frame (Fig. 4) was analyzed for social interaction of WT and KO tester fish. WT test fish were largely observed in the zone “I”, and minimal time was noted in other zones (Fig. 4b, d; Additional file 6: Movie S1). In contrast, *dyrk1aa* KO fish spent significantly less time in zone “I” and comparatively more time in the other zones than their WT counterparts (Fig. 4c, d; Additional file 7: Movie S2). In addition, the total number of transit movements between zones was analyzed to reveal further evidence of impaired social interaction of *dyrk1aa* KO zebrafish (Fig. 4e; Additional file 8: Fig. S6). We hypothesized that the impaired social interaction of *dyrk1aa* KO fish may be due, at least in part, to reduced neuronal function of the brain. To test this idea, we investigated *c-fos* expression, a functional marker of neuronal activation [51], and consistent with our prediction, *c-fos* activation was greatly reduced in the brain of KO fish compared to control WT fish. This reduction was observed in the ventral hypothalamic region (Fig. 5a–d) which is highly activated in the brain of WT zebrafish that are subjected to intense handling stress [51, 52]. Subsequently, we analyzed the expression of various neural markers (Additional file 9: Figure S7) and found a significant change in the expression of *crh* mRNA in a specific brain region of *dyrk1aa* KO fish. The WT and *dyrk1aa* KO fish were subjected to acute social isolation for 24 h before analysis; they had been raised in a group environment. In response to acute social isolation, expression levels of *crh* in KO fish brains of were found to be reduced in the preoptic area (PO, the homolog of the mammalian paraventricular nucleus) of the hypothalamus compared to WT (Fig. 5e–h) [53]. CRH encodes for the stress hormone, corticotrophin-releasing hormone, and is expressed in and secreted by neurons of the paraventricular nucleus (PVN) which links stress-related emotional responses and social interaction behaviors in mammals [54–56].

**Additional file 6: Movie S1.** WT fish in social interaction assay. The WT zebrafish shows social interaction with social cues. (AVI 1335 kb)

**Additional file 7: Movie S2.** KO fish in social interaction assay. The *dyrk1aa* KO zebrafish has no interest in social cues. (AVI 1367 kb)


### Development of a novel shoaling assay in WT zebrafish

**Fig. 6:**
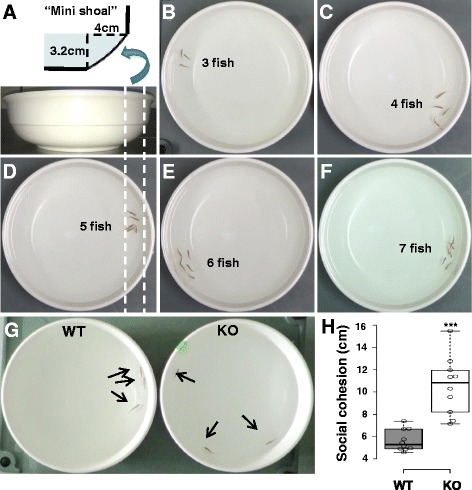
*dyrk1aa* KO zebrafish show impaired social behavior in the shoaling assay. **a** Schematic description of the “mini shoal” formed at the ridge of water body in a flat and round bowl. **b**–**f** Snapshots of shoaling behavior in a group of WT fish, ranging from three to seven fish/group. In most cases, the WT fish group showed “social cohesion” moving together along the narrow space of “mini shoal” in the round bowl. **g** A snapshot of group behavior during the shoaling assay. Three fish were used as a group in this assay. Arrows indicate individual adult fish. **h** Mean distance between individuals (cm) was used to show the degree of social cohesion. The *dyrk1aa* KO fish group showed “loosened” social cohesion, compared to WT fish. Number of trials for this experiment: *n* = 10. Data are presented as mean ± SEM. *** *p* < 0.001 by Student’s *t* test

Zebrafish actively form shoals which are highly sensitive to various experimental manipulations and thus can be used to quantify social behavior [57, 58]. The distances between individual fish can reveal whether they are socially interactive or experiencing impaired social behavior among conspecifics [32, 33]. The degree of shoaling behavior, i.e., social cohesion, was presented by the mean distance (cm) between the individual fish in a group (Additional file 10: Figure S8A). We tested various shoaling experimental conditions using WT fish: differences in the size of the shoaling group (3–7 fish), water volume (1–4 l), and water depth (1.8–5.6 cm). We determined that a group of three fish and a water depth of 3.2 cm in a round bowl with a 24-cm-inside diameter were optimal conditions for the assay (Additional file 10: Figure S8B, C). Notably, the mean distance was relatively constant with an average of 5.8 cm in the WT fish groups. In addition, we observed that fish mostly moved as a group along the narrow ridge of the round bowl (Fig. 6b–f). Since the shape of this small ridge (4-cm width and 3.2-cm depth with curve) looks similar to that of the natural “shoal”, e.g., in stream, we called this area a “mini shoal”.

### *dyrk1aa* KO zebrafish show decreased social cohesion

**Fig. 7:**
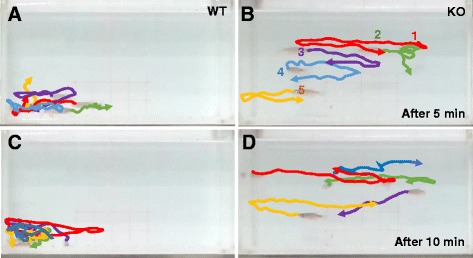
Tracking of individual fish in a group of five fish show impaired social cohesion in KO fish. **a**–**d** Movement of a group of five fish was analyzed after video tracking. The position of individual fish during short periods of 1.5 s at two different time windows (5 and 10 min, respectively) was traced, and their path was presented in different colors (#1 fish in red, #2 fish in green, and so on). Aggregation of WT fish group in a corner of tank is apparent (**a**, **c**), compared to independent free swimming of individual KO fish (**b**, **d**)



We next tested group behavior of *dyrk1aa* KO fish using the shoaling bowl assay. Comparison of *dyrk1aa* KO and WT fish revealed that KO fish group had significantly larger mean distance (10.6 cm, compared to 5.6 cm of WT fish) between each individual fish (Fig. 6g, h; Additional file 11: Movie S3). As a supplemental experiment, we examined group behavior of five fish in a rectangular tank, plotting the path of individual fish after video tracking (Fig. 7a–d; Additional file 12: Movie S4). Social cohesion, aggregation, or shoaling behavior was apparent in the WT fish group; however, the KO fish group showed that individual fish moved independently from one another to suggest deficits in their social interaction.

**Additional file 11: Movie S3.** WT vs KO fish in the shoaling assay. The distance between individual fish in *dyrk1aa* KO fish group is greater than that of WT fish group. (AVI 1035 kb)

**Additional file 12: Movie S4.** WT vs KO fish in group behavior. The *dyrk1aa* KO zebrafish show loose group behavior. (AVI 1141 kb)


## Discussion

Although some functional roles of DYRK1A have been implied in mouse studies [19, 20], so far there have been no reported behavioral studies of adult knockout animals with respect to autism. In this study, we generated a KO zebrafish line for *dyrk1aa* after the discovery of an intragenic microdeletion of *DYRK1A* in an individual with microcephaly and autism. We demonstrated through social behavioral tests that *dyrk1aa* KO zebrafish exhibit social impairments reproductive of human ASD phenotypes.

The *DYRK1A* gene is well conserved in vertebrates, including fish, rodents, and humans. Haploinsufficiency of *DYRK1A* in humans results in microcephaly and ASD [12], while knockout of *Dyrk1a* in mice leads to premature death during early development [18]. In the *dyrk1aa* KO zebrafish, we found similar microcephaly and ASD-like phenotypes, yet the fish were viable without embryonic lethality. This discrepancy may be explained in part by reason of the two orthologous *DYRK1A* genes in zebrafish, *dyrk1aa (NM_001080689)* and *dyrk1ab (NM_001347831)*, caused by whole genome duplication of zebrafish [59]. Thus, we can speculate that *dyrk1ab* may compensate the early lethal phenotype and allow the survival of *dyrk1aa* KO zebrafish into adulthood. We can confirm this possibility by generating a double KO line of both genes in further studies.

Previous murine model studies have been unable to link altered brain structure of *Dyrk1a* dysfunction with social behavior as a direct physiological model of ASD. The structural defect in our *dyrk1aa* zebrafish mutant is reminiscent not only of the *Dyrk1a* mouse, but also of other zebrafish models of autism candidate genes. They display significant structural abnormalities including microcephaly and cell death in anterior structures. Historically, linking these altered physiological states to behavioral deficits has been hampered by two major limitations. First is the paucity of bona fide genetic models for autism in zebrafish. Secondly, reported tracking programs to investigate adult fish behavior in 3D is subject to extensive variability, in large part due to the speed at which multiple fish are moving in three dimensions.

To overcome these limitations, we introduced two social behavioral tests: the social interaction and shoaling assay. In the social interaction assay, we optimized the (a) number of fish, (b) time window of monitoring, and (c) composition of separator material. Zebrafish are active animals and have a wide range of locomotion moving from side to side or from top to bottom in their tank. We found that a group of three fish, rather than 1–2 fish, was ideal for the social cue to facilitate recognition, provide better cueing effect, and elicit stronger interaction of tester fish. Previous work has shown that the ability to view and recognize others is an important factor of social cueing [60–62]. We confirmed these observations by demonstrating that a transparent acrylic plate separator provided better recognition of social cues to tester fish than a metal mesh. Utilizing this assay, we showed that *dyrk1aa* KO zebrafish have impaired social interaction as seen by frequent movements towards the far zones. Taken together, this newly optimized social interaction assay provides a useful means of investigating social interaction of zebrafish models in neurobehavioral disorders.

Next, we developed a novel shoaling assay, called the “shoaling bowl assay”. Shoaling behavior is considered an adaptive and effective natural anti-predatory response, which has been utilized in behavioral analyses in vertebrates [32, 39]. This behavior mimics the tendency of zebrafish to live together and is a robust tool for measuring social behavior of group animals. We showed that the “mini shoal”, formed at the edge of the round bowl, is a preferred location for zebrafish to move together as a group along the narrow space of the shoal. We tested shoaling behavior in different group sizes (3–7 fish). A minimum group of three fish was used for the shoaling assay given that the fish maintained a constant distance between individuals regardless of the size of the group. With a minimum number of animals and a two-dimensional (2D) approach, analysis of social cohesion in a flat round bowl avoids the complexity of group behavior in a three-dimensional (3D) tank which is the current standard [63, 64]. To our knowledge, the altered social cohesion of *dyrk1aa* KO is the first experimental demonstration that shoaling behavior of animals can be regulated by a single gene. The interrelationship between anxiety and social cohesion in animal group behavior will be an interesting topic in further studies since collective animal behavior (huddling, flocking, or shoaling) is a defensive strategy employed by many species in response to predatory threat. Our findings open up a new avenue for the study of this evolutionarily important behavior at the molecular and neural circuit levels.

To understand the molecular mechanisms involved in behavioral alterations of KO fish, we analyzed the expression of various neural markers. Among them, we found significant changes in the expression of *c-fos* and *crh* mRNAs in specific brain regions of *dyrk1aa* KO fish. Neuronal activity of KO fish, as indicated by *c-fos* expression, was lower than that of WT fish in the ventral hypothalamic region during social interaction tests, which suggests that the KO fish brain is less activated by social cues. In addition, *crh* expression level in the PO area of the hypothalamus of KO fish in the acute social isolation test was found to be lower than that of WT, demonstrating low responsiveness to stress in the context of social isolation. In mammals, the hypothalamic region is a known source of stress hormone secretion, such as CRH, and has been shown to be largely involved in social interaction behaviors [65]. Thus, we can conclude that the reduction in the size of the *dyrk1aa* KO fish brain exerts structural changes in the neural circuitry involved in executing of proper behavioral responses to external stress signals which is a vital decision-making aspect of social interactions. In future studies, we plan to examine in further detail the neural circuitry directly involved in *dyrk1aa* function and autism.

In this paper, we have optimized a widely used social interaction test and newly developed the shoaling bowl assay as a convenient method to study group behavior. Furthermore, we showed that these tests can be effectively applied to the study of disease model animals in zebrafish. Together, these data demonstrate that *dyrk1aa* KO zebrafish not only recapitulate the neuroanatomical defects of humans with *DYRK1A* mutations but also exhibit similar hallmarks of impairments in social behavior.

## Conclusions

In this study, we identified a patient with an intragenic deletion in *DYRK1A* exhibiting microcephaly and autism. To validate *DYRK1A* as an autism candidate gene, we generated and characterized a *dyrk1aa* KO zebrafish model using behavioral tests and molecular techniques. *dyrk1aa* KO zebrafish displayed microcephaly with social impairments reproductive of human phenotypes of autism. These results indicate a functional deficiency of *DYRK1A* as an underlying disease mechanism for autism. Our tractable and cost-effective approach provides a useful alternative to the utilization of rodent behavioral models in validating ASD candidate genes; especially, this approach can be used to increase the throughput of much-needed functional modeling for the other candidate autism loci that are being identified by large-scale human genetic studies.

## Additional files

Additional file 1: Figure S1. Characterization of early neural development and larval behavioral tests in WT and *dyrk1aa* KO fish. (A-F) Whole-mount in situ hybridization analysis with various molecular markers at 24 hpf (A, B, lateral view) and 48 hpf (C-F, rostral dorsal view): *sox2*, neural stem cell marker; *neurog1*, neuronal determination marker; and *cyclin D1*, cell proliferation marker. Anterior is to the left. Number of fish used for the analysis: 1) after *sox2* staining and photography, each embryo was genotyped for WT (5/19) and KO homozygote (3/19); 2) for *neurog1*, it was WT (6/16) and KO homozygote (4/16); and 3) for *cyclin D1*, WT (3/16) and KO homozygote (5/16), respectively. (G) Locomotion response to dark flashes in WT and *dyrk1aa* KO larvae at 6 dpf. Movement distance was measured by video tracking analysis (cm per every 10 s). With light-on in 30 s-intervals, zebrafish larvae showed a freezing response (yellow box in the graph). However, they showed a startle response to light-off dark condition (gray box). The number of fish used for this assay: *n* = 14 for WT (+/+), *n* = 25 for heterozygote (+/−), and *n* = 9 for KO homozygote (−/−). (H) Circadian rhythms in WT and *dyrk1aa* KO larvae between 5 and 7 dpf. Circadian rhythms of locomotor activity under LD (day-night) cycles were measured. Both WT and *dyrk1aa* KO larvae display a similar pattern of locomotor activity in daytime or nighttime. The number of fish used for this assay: *n* = 11 for control heterozygote (+/−), and *n* = 13 for KO homozygote (−/−). Data are presented as mean ± SEM. (PDF 1219 kb)

Additional file 2: Figure S2. Comparison of brain size in WT and *dyrk1aa* KO fish. (A) Relative size of brain compartments in KO fish brain was shown as a ratio, compared to those in WT fish brain. Also, body length (cm) of WT and KO fish used for this analysis was constant. Tel, Telencephalon; TeO, Tectum Opticum; CCe, Corpus Cerebelli. Number of dissected brains: *n* = 13 for WT fish and *n* = 13 for KO fish. (B) Percent of TeV space in the total brain. Mean value for the TeV sizes was measured in relative sections of multiple brain samples. Number of fish used for this assay: *n* = 6 for WT fish and *n* = 5 for KO fish. Data are presented as mean ± SEM. * *p* < 0.05, ** *p* < 0.01 by Student’s *t* test. (PDF 1033 kb)

Additional file 3: Figure S3. Analysis of various parameters in the novel tank test. (A, B) Number of entries to the middle or top zone of the tank. (C) Freezing duration. Duration of freezing time (seconds) was measured in every minute. KO zebrafish show reduced freezing behavior, compared to that of WT zebrafish. (D, E) Total distance moved (cm) and mean velocity (cm/s). No significant difference was detected between WT and KO fish. Number of fish used in this assay: *n* = 8 for WT fish, *n* = 8 for KO fish, respectively. Data are presented as mean ± SEM. * *p* < 0.05, ** *p* < 0.01, *** *p* < 0.001 by Student’s *t* test. (PDF 1031 kb)

Additional file 4: Figure S4. Schematic illustration of the social interaction assay with different separation materials. (A-F) Two kinds of materials were used for the separator: metal mesh (A, B, and E) and a clear acrylic plate (C, D, and F). (B, D) Changes of duration time for tester fish, when added to a different number of fish (1–5 fish) as the social cue group, was analyzed in 4 different zones between 6 and 10 min. Multiple trials (*n* = 5) were performed for each group size. (E, F) Detailed information for duration time for tester fish in different zones over the course of 15 min. Data are presented as mean ± SEM. (PDF 1033 kb)

Additional file 5: Figure S5. Analysis of various parameters in social interaction assay. (A, B) Video tracking of WT and *dyrk1aa* KO zebrafish in the social interaction assay. Dashed lines indicate position of a separator and a transparent acrylic plate was used in this experiment. Red lines show tracking of swim movement of WT and KO zebrafish. Fish were tracked every minute for 15 min during the social interaction assay. In this experiment, 3 fish were used as the social cue group in left side and 1 tester fish in right side. (C-F) Duration time for WT and *dyrk1aa* KO zebrafish in each zone; very close zone “I” (C), close zone “II” (D), far zone “III” (E), and very far zone “IV” (F). (G, H) Total distance moved (cm) and mean velocity (cm/s) are no different in both WT and KO fish during the test. Data were collected from video tracking for 15 min and every minute was analyzed. Number of tester fish used in the assay: *n* = 30 for WT and *n* = 30 for KO. Data are presented as mean ± SEM. * *p* < 0.05 by Student’s *t* test. (PDF 1029 kb)

Additional file 8: Figure S6. Number of transit movements of tester fish at each zone boundary. (A-C) 3 cases of each transit movements were analyzed: movement “I” to “II” (A), “II” to “III” (B), and “III” to “IV” zone (C). Number of tester fish used in this assay: *n* = 30 for WT and *n* = 30 for KO. Data are presented as mean ± SEM. * *p* < 0.05 by Student’s *t* test. (PDF 1954 kb)

Additional file 9: Figure S7. Presence of neuromodulator producing cells in the brain of WT and *dyrk1aa* KO zebrafish. (A-H) Expression of *oxt, th, vglut2.2,* and *gad1b* in the brain of WT and *dyrk1aa* KO zebrafish at adult stages. In situ hybridization revealed *dyrk1aa* KO fish show unchanged neuromodulator-producing cells in the brain. (A, B) *oxt*, oxytocinergic marker. (C, D) *vglut2.2*, glutamatergic marker. (E, F) *th1*, dopaminergic marker. (G, H) *gad1b*, GABAergic marker. Anterior to the top and ventral view. Scale bars: 0.2 mm. (PDF 1051 kb)

Additional file 10: Figure S8. Social cohesion in different group sizes and water depths in the shoaling assay. (A) Schematic illustrations of the method to measure the distances between individual fish in the shoaling assay. Different numbers of fish were used as a group, ranging from 3 to 7. Social cohesion was represented as a mean distance of individual fish in the group. (B, C) Mean distance between individuals (cm) in a fish group represents the degree of social cohesion. The mean distance for social cohesion was analyzed in 2 different conditions; 1) different group sizes (B) and 2) water depths and volume (C). Number of trials for each experiment: *n* = 7. Data are presented as mean ± SEM. (PDF 1086 kb)

## Abbreviations

2D: Two-dimension
3D: Three-dimension
ASD: Autism spectrum disorders
CCe: Corpus cerebelli
CGH: Comparative genomic hybridization
CT: Computed tomography
DIL: Diffuse nucleus of the inferior lobe
DLR: Derivative log ratio
dpf: Days post-fertilization
DSCR: Down syndrome critical region
Fb: Forebrain
Hb: Hindbrain
hpf: Hours post-fertilization
Hy: Hypothalamus
ISCA: International standard cytogenomic array
KO: Knockout
LD: Light-dark
Mb: Midbrain
mpf: Months post-fertilization
MRI: Magnetic resonance imaging
PFA: Paraformaldehyde
PO: Preoptic area
PVN: Paraventricular nucleus
SEM: Standard error of the mean
TALEN: Transcription activator-like effector nuclease
Tel: Telencephalon
TeO: Tectum opticum
TeV: Tectal ventricle
WT: Wild type
