# Supplementary figures and images for: Zebrafish knockout of Down syndrome gene, DYRK1A, shows social impairments relevant to autism
Source: Mol Autism. 2017 Sep 29;8:50. doi: 10.1186/s13229-017-0168-2 (PMC5622473; doi:10.1186/s13229-017-0168-2)

Additional file 1: Figure S1

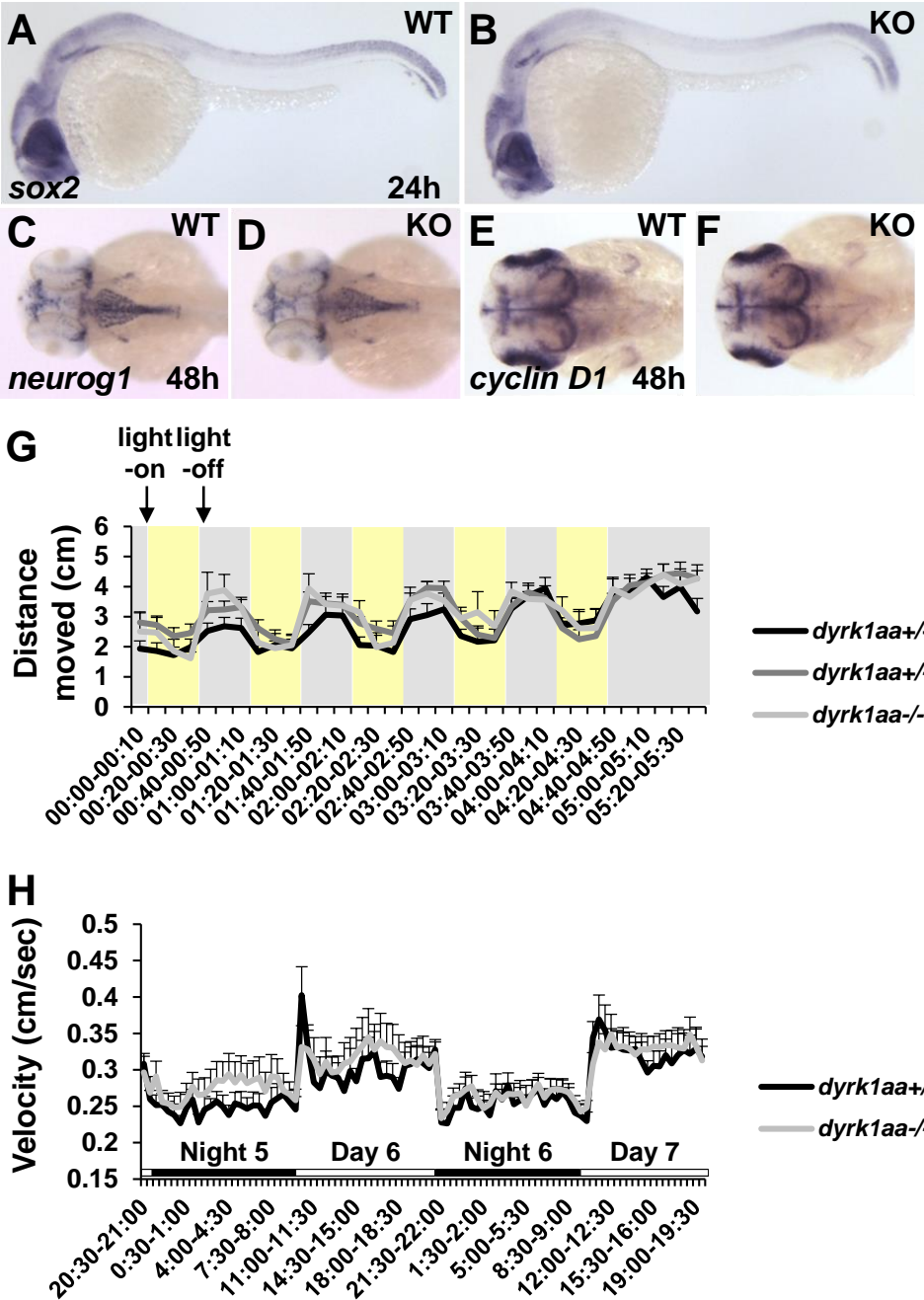

Supplement: Supplementary file 5 — Characterization of early neural development and larval behavioral tests in WT and dyrk1aa KO fish. (A-F) Whole-mount in situ hybridization analysis with various molecular markers at 24 hpf (A, B, lateral view) and 48 hpf (C-F, rostral dorsal view): sox2, neural stem cell marker; neurog1, neuronal determination marker; and cyclin D1, cell proliferation marker. Anterior is to the left. Number of fish used for the analysis: 1) after sox2 staining and photography, each embryo was genotyped for WT (5/19) and KO homozygote (3/19); 2) for neurog1, it was WT (6/16) and KO homozygote (4/16); and 3) for cyclin D1, WT (3/16) and KO homozygote (5/16), respectively. (G) Locomotion response to dark flashes in WT and dyrk1aa KO larvae at 6 dpf. Movement distance was measured by video tracking analysis (cm per every 10 s). With light-on in 30 s-intervals, zebrafish larvae showed a freezing response (yellow box in the graph). However, they showed a startle response to light-off dark condition (gray box). The number of fish used for this assay: n = 14 for WT (+/+), n = 25 for heterozygote (+/−), and n = 9 for KO homozygote (−/−). (H) Circadian rhythms in WT and dyrk1aa KO larvae between 5 and 7 dpf. Circadian rhythms of locomotor activity under LD (day-night) cycles were measured. Both WT and dyrk1aa KO larvae display a similar pattern of locomotor activity in daytime or nighttime. The number of fish used for this assay: n = 11 for control heterozygote (+/−), and n = 13 for KO homozygote (−/−). Data are presented as mean ± SEM. (PDF 1219 kb) [file 13229_2017_168_MOESM1_ESM.pdf]

# Additional file 2: Figure S2

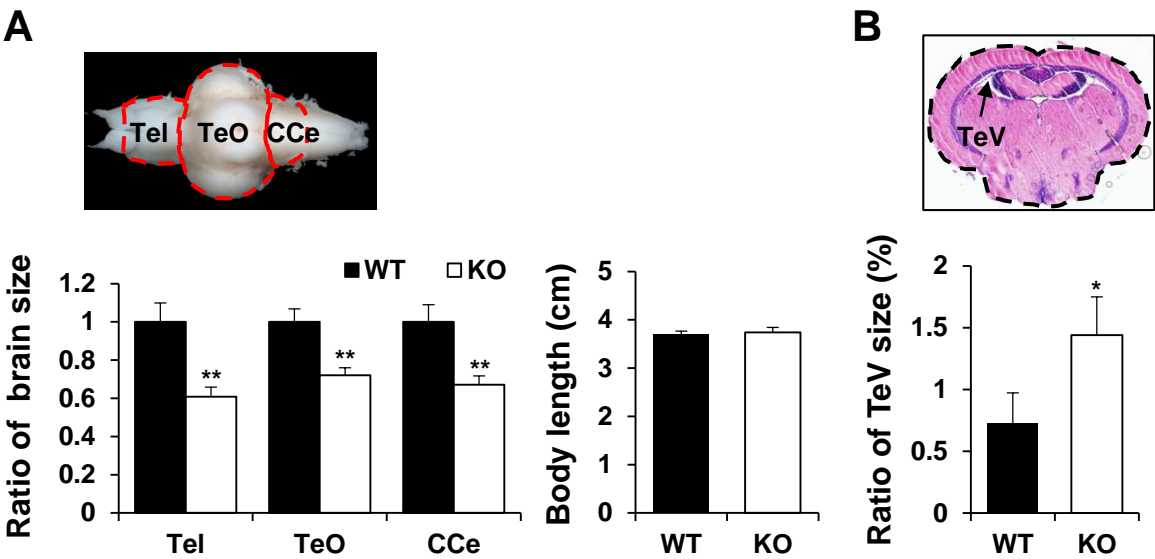

Supplement: Supplementary file 6 — Comparison of brain size in WT and dyrk1aa KO fish. (A) Relative size of brain compartments in KO fish brain was shown as a ratio, compared to those in WT fish brain. Also, body length (cm) of WT and KO fish used for this analysis was constant. Tel, Telencephalon; TeO, Tectum Opticum; CCe, Corpus Cerebelli. Number of dissected brains: n = 13 for WT fish and n = 13 for KO fish. (B) Percent of TeV space in the total brain. Mean value for the TeV sizes was measured in relative sections of multiple brain samples. Number of fish used for this assay: n = 6 for WT fish and n = 5 for KO fish. Data are presented as mean ± SEM. * p < 0.05, ** p < 0.01 by Student’s t test. (PDF 1033 kb) [file 13229_2017_168_MOESM2_ESM.pdf]

Additional file 3: Figure S3

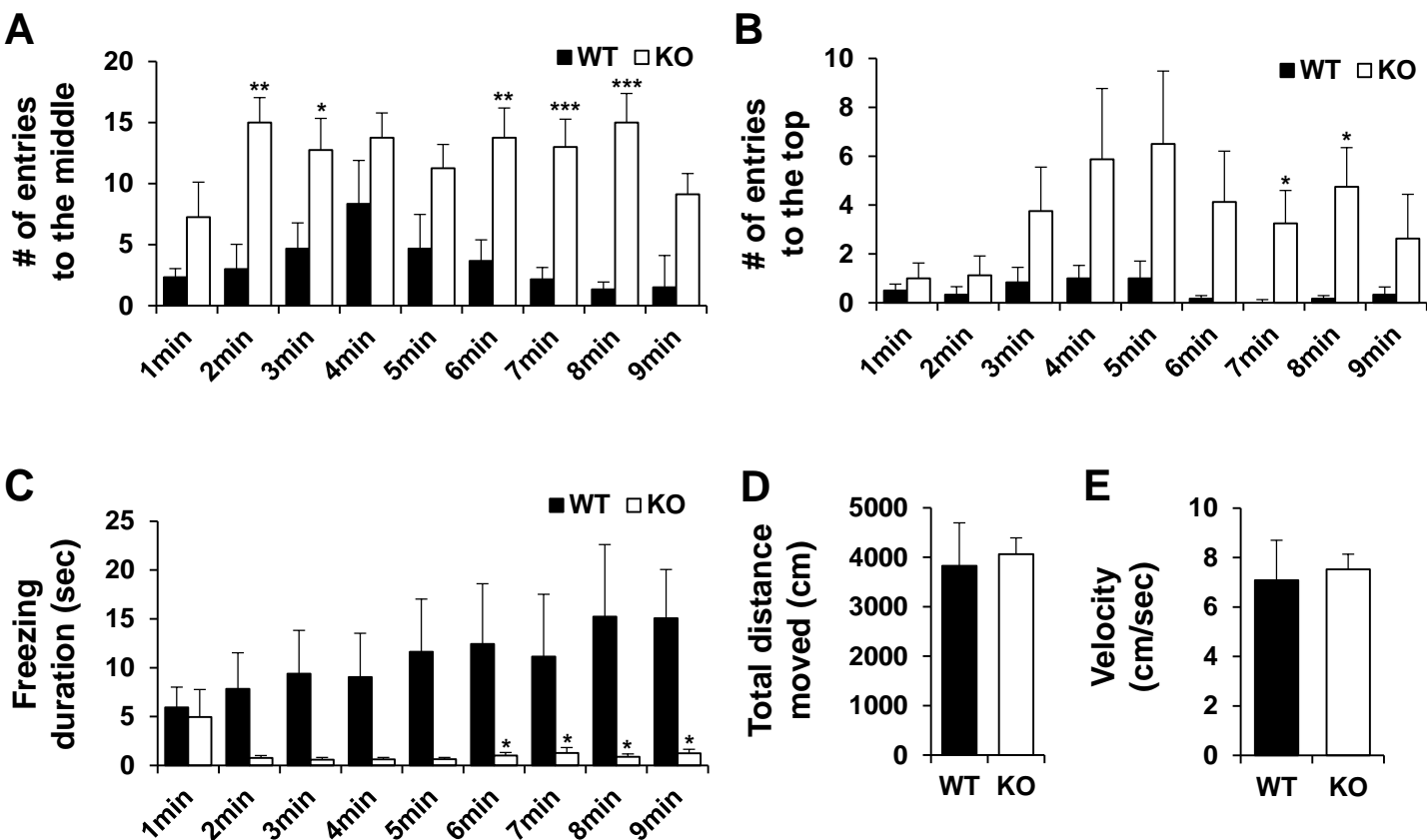

Supplement: Supplementary file 7 — Analysis of various parameters in the novel tank test. (A, B) Number of entries to the middle or top zone of the tank. (C) Freezing duration. Duration of freezing time (seconds) was measured in every minute. KO zebrafish show reduced freezing behavior, compared to that of WT zebrafish. (D, E) Total distance moved (cm) and mean velocity (cm/s). No significant difference was detected between WT and KO fish. Number of fish used in this assay: n = 8 for WT fish, n = 8 for KO fish, respectively. Data are presented as mean ± SEM. * p < 0.05, ** p < 0.01, *** p < 0.001 by Student’s t test. (PDF 1031 kb) [file 13229_2017_168_MOESM3_ESM.pdf]

Additional file 4: Figure S4

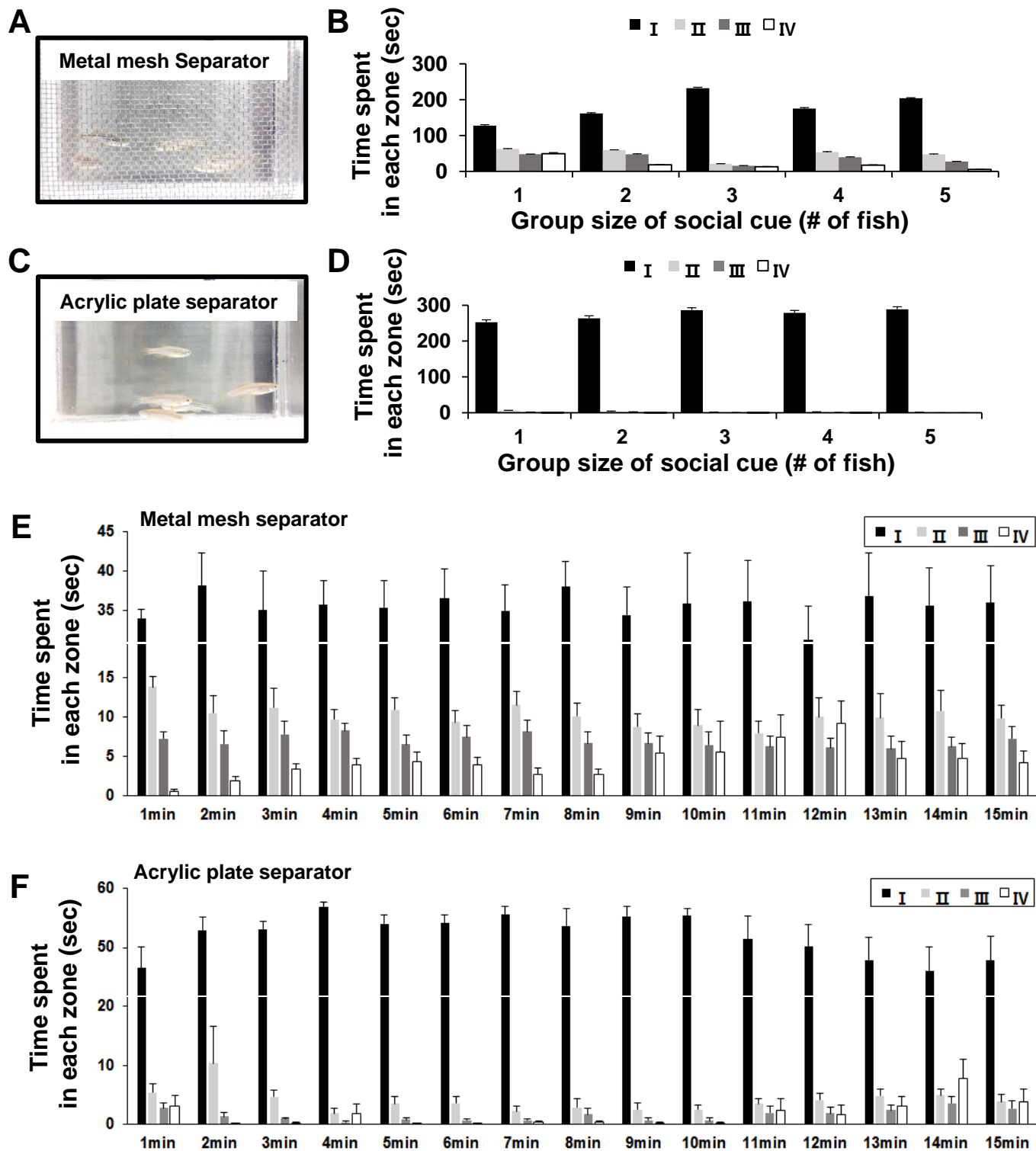

Supplement: Supplementary file 8 — Schematic illustration of the social interaction assay with different separation materials. (A-F) Two kinds of materials were used for the separator: metal mesh (A, B, and E) and a clear acrylic plate (C, D, and F). (B, D) Changes of duration time for tester fish, when added to a different number of fish (1–5 fish) as the social cue group, was analyzed in 4 different zones between 6 and 10 min. Multiple trials (n = 5) were performed for each group size. (E, F) Detailed information for duration time for tester fish in different zones over the course of 15 min. Data are presented as mean ± SEM. (PDF 1033 kb) [file 13229_2017_168_MOESM4_ESM.pdf]

Additional file 5: Figure S5

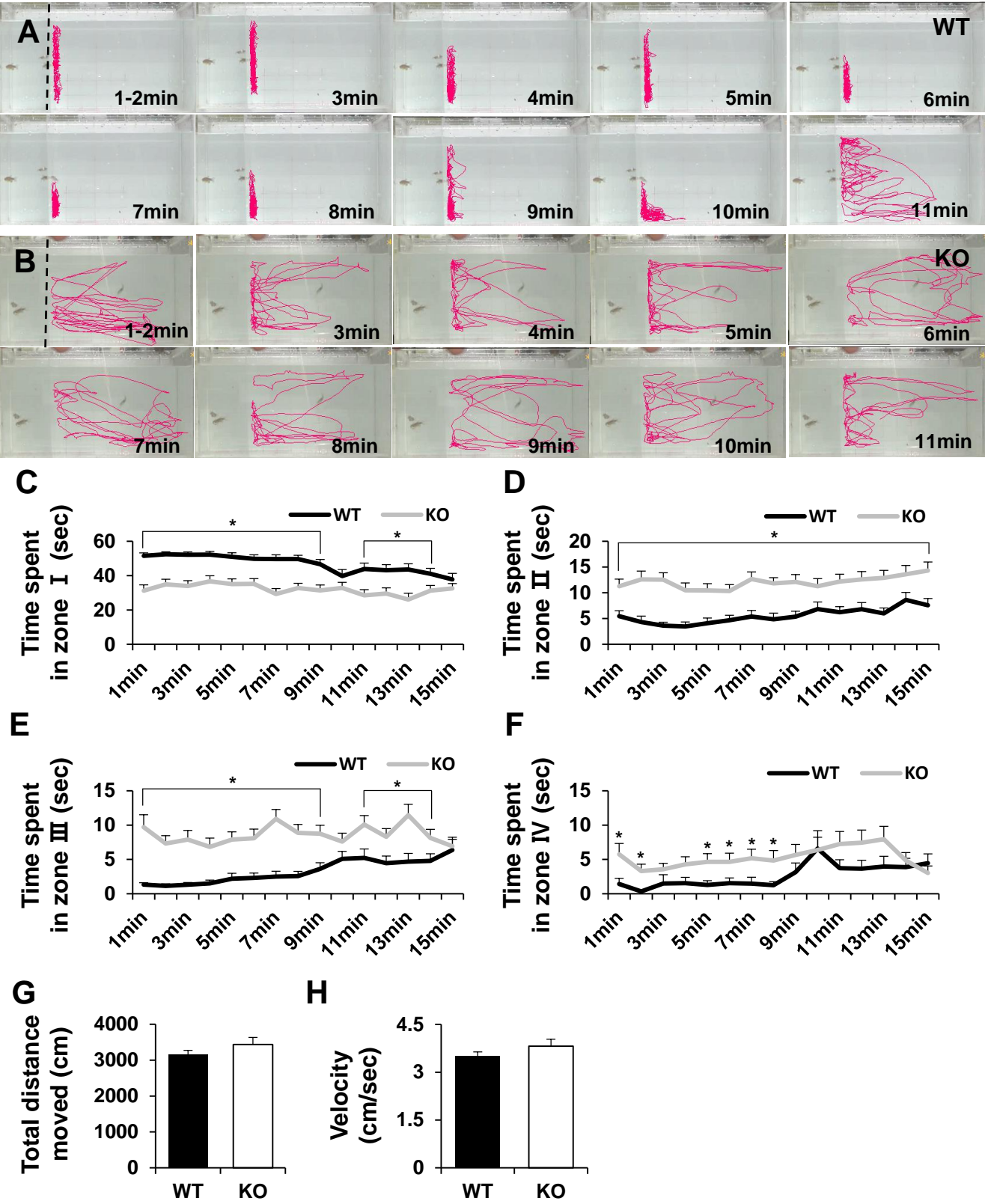

Supplement: Supplementary file 9 — Analysis of various parameters in social interaction assay. (A, B) Video tracking of WT and dyrk1aa KO zebrafish in the social interaction assay. Dashed lines indicate position of a separator and a transparent acrylic plate was used in this experiment. Red lines show tracking of swim movement of WT and KO zebrafish. Fish were tracked every minute for 15 min during the social interaction assay. In this experiment, 3 fish were used as the social cue group in left side and 1 tester fish in right side. (C-F) Duration time for WT and dyrk1aa KO zebrafish in each zone; very close zone “I” (C), close zone “II” (D), far zone “III” (E), and very far zone “IV” (F). (G, H) Total distance moved (cm) and mean velocity (cm/s) are no different in both WT and KO fish during the test. Data were collected from video tracking for 15 min and every minute was analyzed. Number of tester fish used in the assay: n = 30 for WT and n = 30 for KO. Data are presented as mean ± SEM. * p < 0.05 by Student’s t test. (PDF 1029 kb) [file 13229_2017_168_MOESM5_ESM.pdf]

Additional file 6: Figure S6

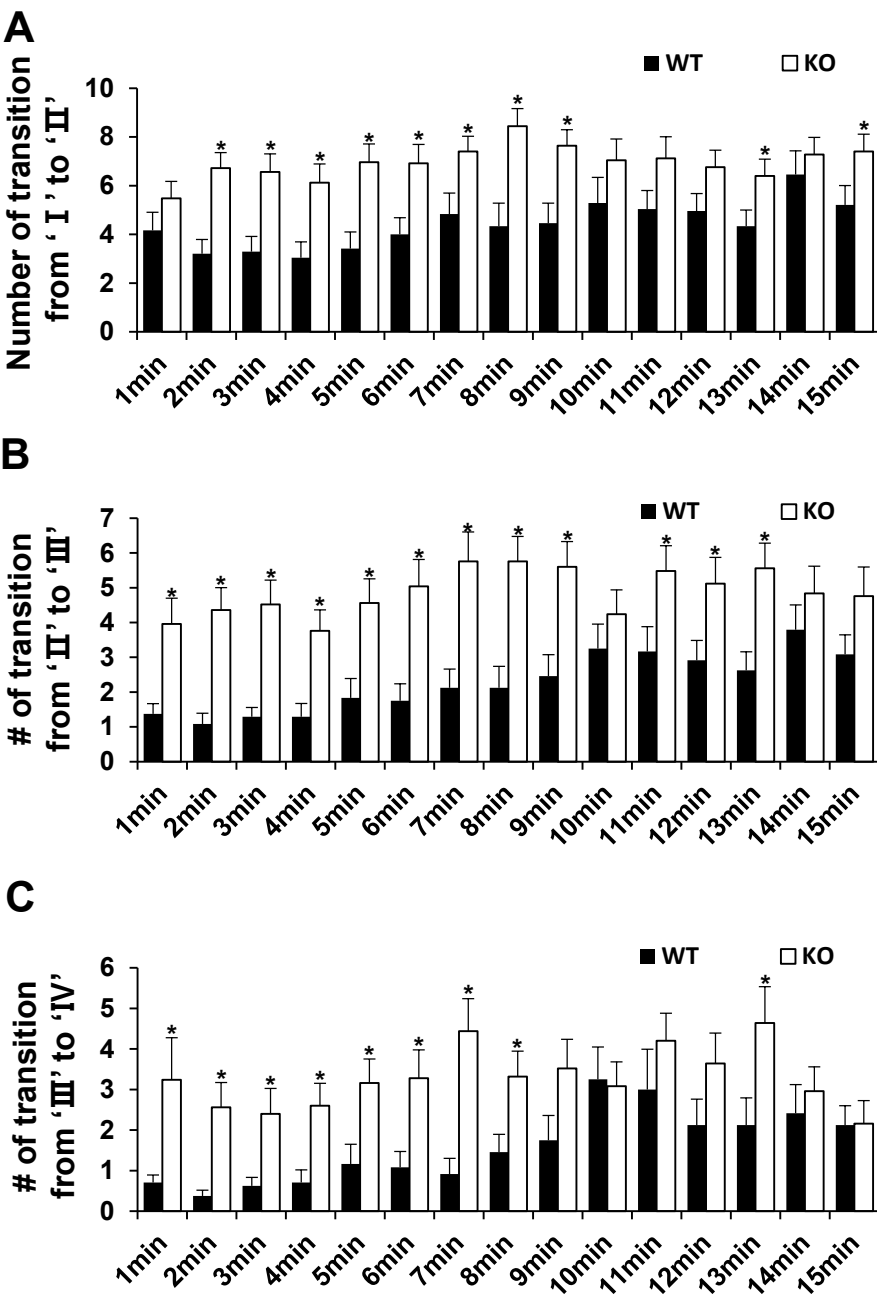

Supplement: Supplementary file 10 — Number of transit movements of tester fish at each zone boundary. (A-C) 3 cases of each transit movements were analyzed: movement “I” to “II” (A), “II” to “III” (B), and “III” to “IV” zone (C). Number of tester fish used in this assay: n = 30 for WT and n = 30 for KO. Data are presented as mean ± SEM. * p < 0.05 by Student’s t test. (PDF 1954 kb) [file 13229_2017_168_MOESM8_ESM.pdf]

Additional file 7: Figure S7

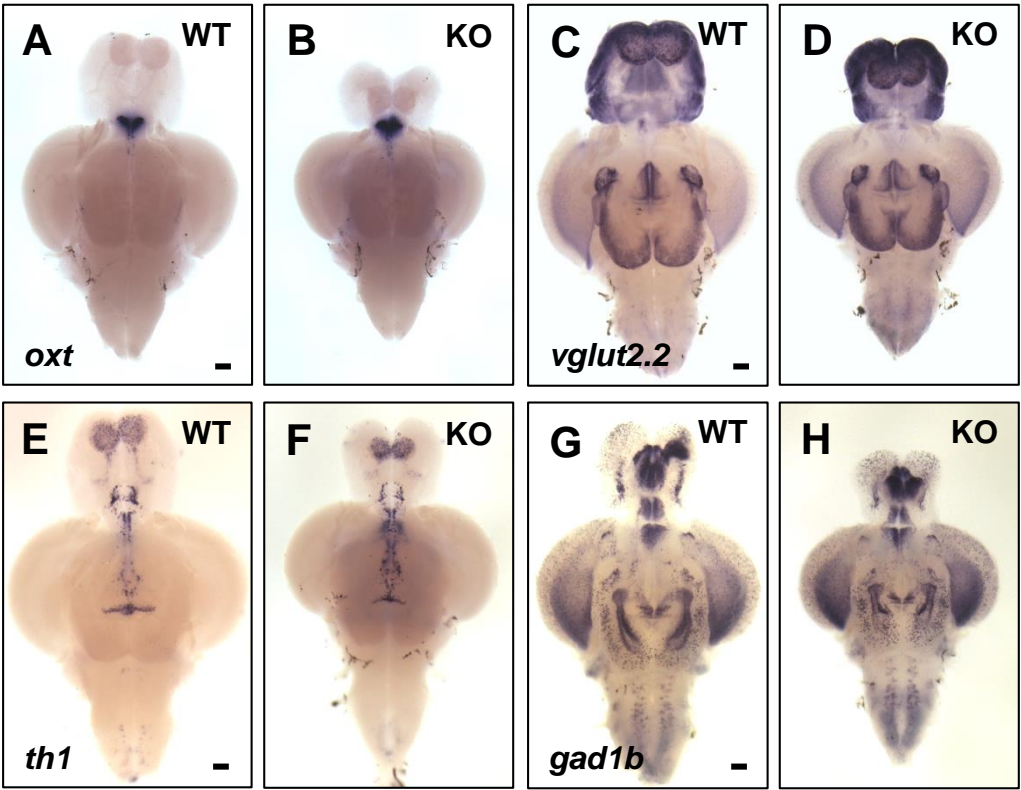

Supplement: Supplementary file 11 — Presence of neuromodulator producing cells in the brain of WT and dyrk1aa KO zebrafish. (A-H) Expression of oxt, th, vglut2.2, and gad1b in the brain of WT and dyrk1aa KO zebrafish at adult stages. In situ hybridization revealed dyrk1aa KO fish show unchanged neuromodulator-producing cells in the brain. (A, B) oxt, oxytocinergic marker. (C, D) vglut2.2, glutamatergic marker. (E, F) th1, dopaminergic marker. (G, H) gad1b, GABAergic marker. Anterior to the top and ventral view. Scale bars: 0.2 mm. (PDF 1051 kb) [file 13229_2017_168_MOESM9_ESM.pdf]

Additional file 8: Figure S8

A

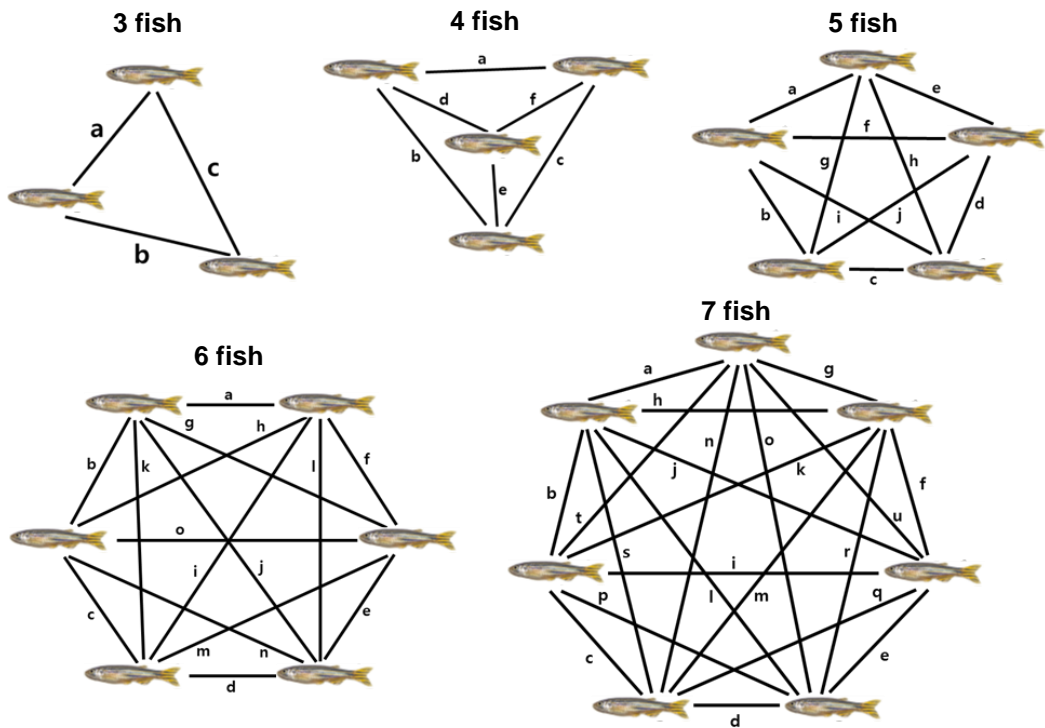

B

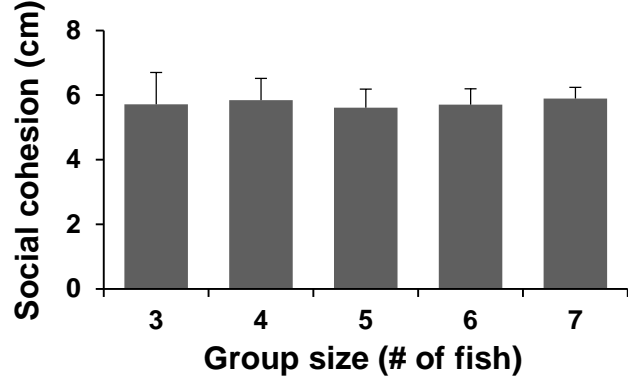

C

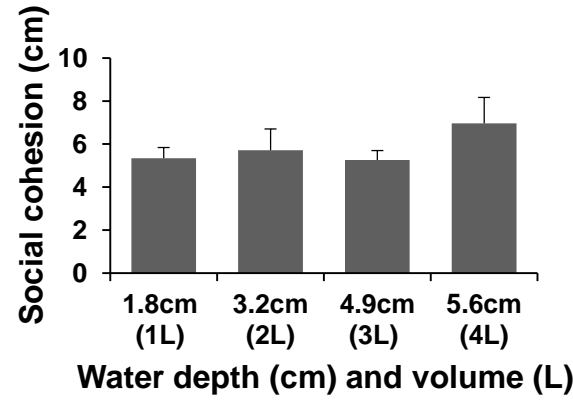

Supplement: Supplementary file 12 — Social cohesion in different group sizes and water depths in the shoaling assay. (A) Schematic illustrations of the method to measure the distances between individual fish in the shoaling assay. Different numbers of fish were used as a group, ranging from 3 to 7. Social cohesion was represented as a mean distance of individual fish in the group. (B, C) Mean distance between individuals (cm) in a fish group represents the degree of social cohesion. The mean distance for social cohesion was analyzed in 2 different conditions; 1) different group sizes (B) and 2) water depths and volume (C). Number of trials for each experiment: n = 7. Data are presented as mean ± SEM. (PDF 1086 kb) [file 13229_2017_168_MOESM10_ESM.pdf]
